# Identification of the Kelch Family Protein Nd1-L as a Novel Molecular Interactor of KRIT1

**DOI:** 10.1371/journal.pone.0044705

**Published:** 2012-09-06

**Authors:** Paolo Guazzi, Luca Goitre, Elisa Ferro, Valentina Cutano, Chiara Martino, Lorenza Trabalzini, Saverio Francesco Retta

**Affiliations:** 1 Department of Biotechnologies, Chemistry and Pharmacy, University of Siena, Siena, Italy; 2 Department of Clinical and Biological Sciences, University of Torino, Orbassano (TO), Italy; St. Georges University of London, United Kingdom

## Abstract

Loss-of-function mutations of the *KRIT1* gene (*CCM1*) have been associated with the Cerebral Cavernous Malformation (CCM) disease, which is characterized by serious alterations of brain capillary architecture. The KRIT1 protein contains multiple interaction domains and motifs, suggesting that it might act as a scaffold for the assembly of functional protein complexes involved in signaling networks. In previous work, we defined structure-function relationships underlying KRIT1 intramolecular and intermolecular interactions and nucleocytoplasmic shuttling, and found that KRIT1 plays an important role in molecular mechanisms involved in the maintenance of the intracellular Reactive Oxygen Species (ROS) homeostasis to prevent oxidative cellular damage. Here we report the identification of the Kelch family protein Nd1-L as a novel molecular interactor of KRIT1. This interaction was discovered through yeast two-hybrid screening of a mouse embryo cDNA library, and confirmed by pull-down and co-immunoprecipitation assays of recombinant proteins, as well as by co-immunoprecipitation of endogenous proteins in human endothelial cells. Furthermore, using distinct KRIT1 isoforms and mutants, we defined the role of KRIT1 domains in the Nd1-L/KRIT1 interaction. Finally, functional assays showed that Nd1-L may contribute to the regulation of KRIT1 nucleocytoplasmic shuttling and cooperate with KRIT1 in modulating the expression levels of the antioxidant protein SOD2, opening a novel avenue for future mechanistic studies. The identification of Nd1-L as a novel KRIT1 interacting protein provides a novel piece of the molecular puzzle involving KRIT1 and suggests a potential functional cooperation in cellular responses to oxidative stress, thus expanding the framework of molecular complexes and mechanisms that may underlie the pathogenesis of CCM disease.

## Introduction

KRIT1 is a gene responsible for Cerebral Cavernous Malformations (CCM), a major cerebrovascular disease, with a prevalence of 0.1%–0.5% in the general population, characterized by abnormal vascular sinusoids that predispose to seizures, focal neurological deficits, and fatal intracerebral hemorrhage [1], [2], [3], [4]. Comprehensive analysis of the KRIT1 gene in CCM patients has suggested that KRIT1 functions need to be severely impaired for pathogenesis [5], [6], [7]. On the other hand, the targeted disruption of *KRIT1* gene in animal models has resulted in lethal vascular defects early in embryogenesis, pointing to a major role for the KRIT1 protein in blood vessel formation/stabilization [8]. In addition, our recent data demonstrate that KRIT1 plays an important role in molecular mechanisms involved in the maintenance of the intracellular Reactive Oxygen Species (ROS) homeostasis to prevent oxidative cellular damage, suggesting a novel mechanism for CCM pathogenesis [9].

Nevertheless, the physio-pathological role of KRIT1 is still elusive and remains a fundamental research challenge for understanding the molecular bases of the CCM pathogenesis.

KRIT1 lacks defined catalytic domains while contains well characterized protein-protein interaction motifs and domains, including three NPXY/F motifs, three ankyrin repeats and a FERM domain, indicating a possible function as scaffold protein for organizing protein complexes involved in signaling networks [10], [11], [12], [13], [14], [15], [16].

The primary insight into KRIT1 function came from its original isolation in a yeast two-hybrid (Y2H) screening as a binding partner of the small GTPase Rap1 [12], a Ras family member involved in fundamental physiological processes through the regulation of cadherin- and integrin-dependent cell adhesion and signaling [17], [18]. Specifically, the interaction with Rap1 has been shown to occur at the KRIT1 C-terminal FERM domain, a clover-shaped structural module formed by three subdomains (F1, F2 and F3) which is known to mediate intermolecular interactions with partner proteins and phospholipids as well as intramolecular regulatory interactions [19]. Indeed, subsequent researches demonstrated that the interaction of KRIT1 with Rap1 plays an important role in stabilization of endothelial cell-cell junctions and regulation of cell-cell adhesion processes in both endothelial and non-endothelial tissues [20], [21].

Additional clues to the function of KRIT1 have been provided by the demonstration of other molecular interactions mediated by distinct domains of the protein.

In particular, a NPXY motif located in the N-terminal region of KRIT1 (aa 1–207) is required for the interaction with ICAP1, a PhosphoTyrosine-Binding (PTB) domain-containing protein previously characterized as interactor and regulator of β1 integrin, suggesting that KRIT1 may regulate the ICAP1 regulatory effect on β1 integrin [13], [15].

Two additional NPXY/F motifs located in the central region of KRIT1 (aa 208–417) have been demonstrated to be required for KRIT1 interaction with CCM2 [14], [16], and subsequently it has been shown that through this interaction KRIT1 regulates vascular permeability by suppressing the RhoA-ROCK signaling pathway, strongly dysregulated in human CCM endothelium [22], [23]. Furthermore, we recently demonstrated that the region containing the second and third NPXY/F motifs is involved in a head-to-tail intramolecular interaction with the PTB-like F3 subdomain of the C-terminal FERM domain and suggested a model where KRIT1A behaves as a molecular switch and regulates its function by adopting different open/closed conformations. This open/closed conformation switch mediates mutually exclusive effects on KRIT1A nuclear translocation and molecular interactions [11].

On the other hand, we previously identified a second KRIT1 isoform, KRIT1B, characterized by the alternative splicing of the 15th coding exon, which results in a shorter protein lacking 39 aa in the C-terminus [24]. We found that the alternative splicing strongly affects the structure of the F3/PTB lobe of the KRIT1 FERM domain with important functional implications, including the loss of the ability to undertake head-to-tail intramolecular interaction, binding to Rap1 and nucleocytoplasmic shuttling.

KRIT1 also contains three ankyrin repeats located between the NPXY/F motifs and the FERM domain. Although ankyrin repeats are found in a multitude of proteins of diverse functions and are known to mediate protein-protein interactions [25], no binding proteins for the KRIT1 ankyrin repeats have been identified so far.

The presence of distinct binding motifs and domains strongly indicates that KRIT1 can interact with multiple protein partners to regulate complex signaling pathways, suggesting that the identification of novel molecular interactors may represent a crucial step in elucidating its function.

To further define KRIT1 molecular and cellular functions and provide novel insights for a better understanding of CCM pathogenesis mechanisms, we attempted to find new KRIT1 interaction partners using a Y2H screening approach. Here we report the identification and characterization of a novel KRIT1 molecular interaction with Nd1-L, a member of the BTB/Kelch protein family.

## Results

### Identification of Nd1 as a novel interaction partner of KRIT1

**Figure 1:**
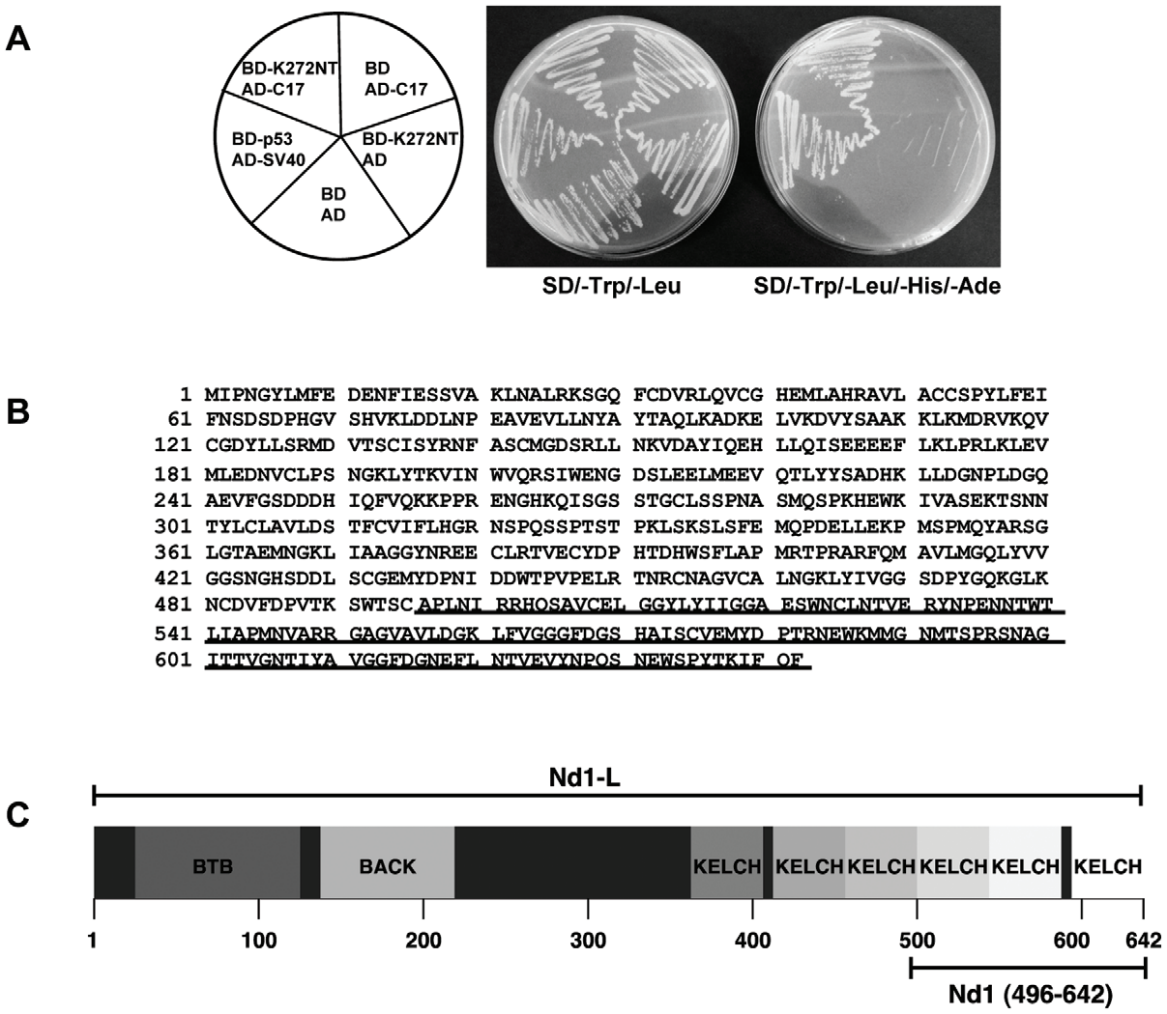
Identification of Nd1-L as a new interaction partner of KRIT1. **A.** Small scale yeast two-hybrid analysis of the interaction of KRIT1 with the C17 clone. Yeast strain AH109 was cotransformed with GAL4DNA-BD and GAL4AD fusion constructs as indicated (left). Cotransformed AH109 cells were streaked out on plates lacking tryptophan and leucine (SD/-Trp/-Leu) or plates lacking tryptophan, leucine, histidine and adenine (SD/-Trp/-Leu/-His/-Ade). The known interaction between p53 protein and SV40 large T-antigen was used as positive control. Empty vectors encoding the GAL4DNA-BD and AD were used as negative control and to exclude aspecific interactions of both K272NT and C17. **B.** Amino acid sequence of Nd1-L (NP_001034601.1). The sequence of C17 corresponding to Nd1-L (496–642) is underlined. **C.** Domain structure of the Nd1-L protein. The fragment identified as K272NT interactor in the yeast two-hybrid assay, Nd1-L (496–642), contains a portion of the kelch domain extending from the C-terminal portion of the third to the sixth kelch repeat motif.

In order to identify new binding partners of KRIT1 we used the GAL4BD-K272NT fusion construct, encoding the N-terminal 272 aa fragment of KRIT1 fused to the GAL4 DNA binding domain, as bait for the Y2H screening of a mouse embryo cDNA library. Twenty-two clones positive by *HIS3, ADE2, MEL1* and *LACZ* expression were initially isolated out of 8.8×10^7^ independent clones. The pACT2 plasmids containing the cDNA sequence of putative KRIT1 interaction partners were recovered from the yeast cells and then used in a small scale two hybrid assay to verify the specific interaction with KRIT1. Among the 22 isolated clones, 5 exhibited a K272NT-dependent *HIS3/ADE2*-positive genotype upon retransformation. DNA sequencing and Genebank database search revealed that one of these five clones, named C17 (Fig. 1A), encoded a 147-amino acid polypeptide corresponding to the C-terminal portion (aa 496–642) of the Nd1-L protein (NP_001034601.1) (Fig. 1B) [26]. This is a member of the Kelch family proteins and is characterized by a BTB/POZ domain in its N-terminus (aa 1–128) followed by a BACK domain (aa 134–233) and six canonical kelch repeat motifs at the C-terminus.

The Nd1-L fragment encoded by the C17 clone (Nd1-L 496–642) is devoid of the BTB/POZ and BACK domains, containing only a portion of the kelch repeats comprised from the C-terminal portion of the third to the sixth motif (Fig. 1C).

### Nd1-L interacts with KRIT1 both *in vitro* and in endothelial cells

**Figure 2:**
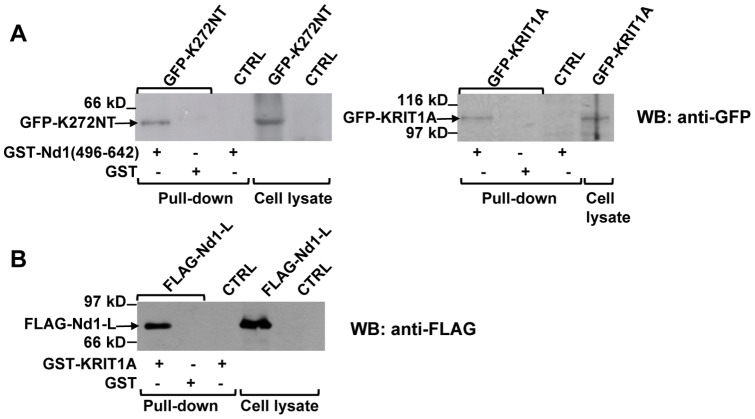
The Nd1-L protein interacts with KRIT1A *in vitro*. **A.** HEK293 cells were transiently transfected with pEGFP vector containing K272NT (left panel) or full-length KRIT1A (right panel). The pull-down assay was performed by incubating transfected (GFP-K272NT, GFP-KRIT1A) or mock-transfected (CTRL) cell lysates with GST-Nd1-L (496–642) immobilized on glutathione-sepharose resin, as indicated. Incubation of transfected cell lysates with GST-glutathione-sepharose was used as negative control. The different incubation mixtures were then analyzed by SDS-PAGE and western blotting with anti-GFP together with aliquots of transfected and mock-transfected cell lysates as controls. **B.** HEK293 cells were transiently transfected with 2FLAG-Nd1-L. Lysates from transfected (FLAG-Nd1-L) or mock-transfected (CTRL) cells were then incubated with GST-KRIT1A-glutathione-sepharose or with GST-glutathione-sepharose, as indicated. The different incubation mixtures and aliquots of transfected and mock-transfected cells were analyzed by SDS-PAGE and western blotting with anti-FLAG antibodies.

To confirm the novel interaction identified by the Y2H analysis, a GST pull-down assay of whole-cell extracts from HEK293 cells overexpressing GFP-K272NT or GFP-KRIT1A was performed using a GST-Nd1-L (496–642) fusion protein as a bait. GST-bound fractions were subjected to western blot analysis with anti-GFP antibody. As shown in Fig. 2A, both K272NT and KRIT1A were specifically detected only in the GST-Nd1-L-bound fractions. Thus, consistently with the two-hybrid outcomes, the GST pull-down assay showed that the Nd1-L (496–642) polypeptide encoded by the C17 clone was able to interact with the N-terminal K272NT fragment *in vitro*. In addition, Nd1-L (496–642) interacted also with full-length KRIT1A.

A further pull-down experiment was performed using GST-KRIT1A as bait and a lysate of HEK293 cells overexpressing FLAG-Nd1-L as prey. As shown in Fig. 2B, full-length Nd1-L maintained the capability to interact with KRIT1A *in vitro*.

Control pull-down assays of cell lysates overexpressing the GFP or the FLAG tags was performed to assess the specificity of the interaction between KRIT1 and Nd1.

**Figure 3:**
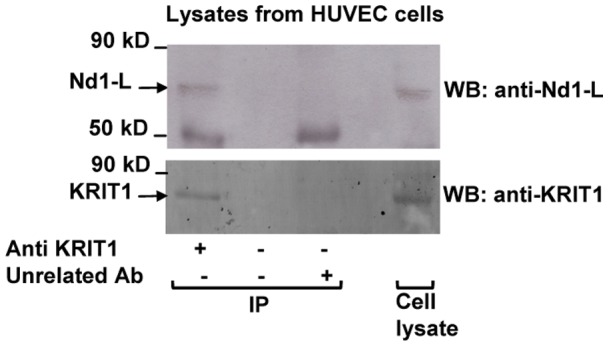
Nd1-L and KRIT1 associate in HUVEC cells. Lysates from HUVEC cells were immunoprecipitated with anti-KRIT1 polyclonal antibody or an unrelated antibody as negative control followed by SDS-PAGE and western blotting with an anti-Nd1 antibody (upper panel). Immunoreactive bands at 50 kDa indicate IgG-heavy chains. The IP of KRIT1 was verified by blotting against KRIT1 (lower panel). An aliquot of the HUVEC cells lysate was loaded in the same gel as positive control for the immunodetection phase and to evaluate the relative expression levels of KRIT1 and Nd1.

In order to demonstrate the interaction between KRIT1A and Nd1-L *in vivo*, we performed co-IP of the two endogenous proteins from human cells. As shown in Fig. 3, endogenous Nd1-L and KRIT1 co-immunoprecipitated from HUVEC cell lysates assayed with an anti-KRIT1 antibody. These results confirmed and extended Y2H and GST pull-down outcomes, demonstrating that the interaction between endogenous KRIT1 and Nd1-L proteins occurs in human endothelial cells.

### Mapping of the KRIT1/Nd1-L interaction

In a previous work we identified a novel KRIT1 isoform, KRIT1B, characterized by the alternative splicing of the 15th coding exon, which results in a 117-bp in-frame deletion accounting for the exclusion of 39 aa in a region close to C-terminus of full-length KRIT1A [24]. The alternative splicing affects the β sandwich of the F3/PTB lobe of the KRIT1 FERM domain. This significant structural impact has important functional implications as it regulates crucial functions of KRIT1, including its head-to-tail intramolecular interaction and nucleocytoplasmic shuttling, Rap1 binding, and modulation of ICAP1 nucleocytoplasmic trafficking [11].

**Figure 4:**
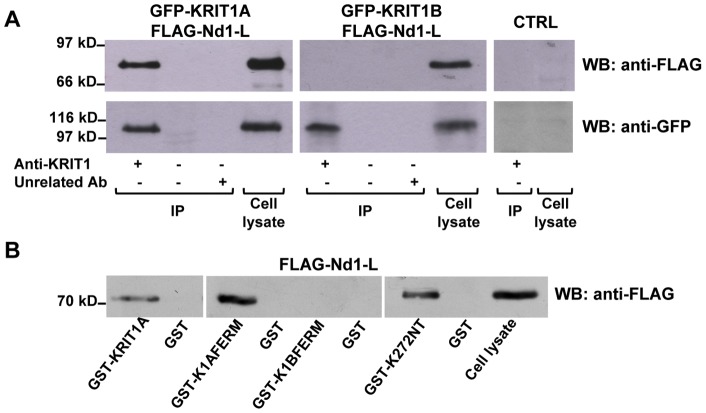
The integrity of the KRIT1 FERM domain is required for the interaction with Nd1-L. A. KRIT1A but not KRIT1B is able to interact with Nd1-L. HEK293 cells were co-transfected with pCR-2FLAG-Nd1-L and pEGFP-KRIT1A or pEGFP-KRIT1B constructs as indicated. 36 hours after co-transfections the cells were harvested and lysed as detailed in Materials and Methods. The co-IP was performed by incubation with anti-KRIT1 or an unrelated antibody as negative control, followed by SDS-PAGE analysis and western blotting with anti-FLAG (upper panel). Mock-transfected cells were used in the assay as controls (CTRL). The IP of EGFP-KRIT1A or EGFP-KRIT1B was verified by anti-GFP western blot (lower panel). Lysates from both transfected and mock-transfected HEK293 cells were loaded in the same gels as positive and negative controls for the immunodetection phase. **B.** Two different regions of KRIT1 are involved in the interaction with Nd1-L. Full-length KRIT1A, K1AFERM, K1BFERM and K272NT were expressed as GST-fusion proteins and ligated to glutathione-sepharose. Lysates from HEK293 cells overexpressing 2FLAG-Nd1-L were incubated with the different affinity resins as indicated. Incubation with GST-glutathione-sepharose was used as negative control. The different incubation mixtures and an aliquot of the transfected cell lysate were analyzed by SDS-PAGE and anti-FLAG western blotting.

To evaluate whether an intact KRIT1 FERM domain was required for the interaction with ND1-L, we used HEK293 cells overexpressing both FLAG-Nd1-L and GFP-KRIT1A or GFP-KRIT1B to perform a co-IP assay. Cell lysates were incubated with the anti-KRIT1 antibody to immunoprecipitate KRIT1-containing complexes and then analyzed by western blotting with an anti-FLAG antibody (Fig. 4A, upper panel). The IP of GFP-KRIT1A and GFP-KRIT1B was verified by probing western blots with the anti-GFP antibody (Fig. 4A, lower panel). Only KRIT1A, but not KRIT1B, was able to co-immunoprecipitate Nd1-L, clearly indicating that an intact FERM domain is required for the interaction of full length KRIT1 with Nd1-L.

In order to further define the involvement of the KRIT1A FERM domain in the interaction with Nd1-L, we performed a GST pull-down assay by probing HEK293 cell lysates overexpressing FLAG-Nd1-L with the FERM domain of KRIT1A and KRIT1B fused to GST (GST-K1AFERM and GST-K1BFERM, respectively).

GST-KRIT1A and GST-K272NT were introduced in the assay as positive controls. As shown in Fig. 4B, K1AFERM, but not K1BFERM, was able to interact with Nd1-L in the pull-down assay, indicating that the KRIT1A FERM domain is involved in the interaction with Nd1-L, and further supporting the finding that the integrity of the FERM domain is required for the interaction.

**Figure 5:**
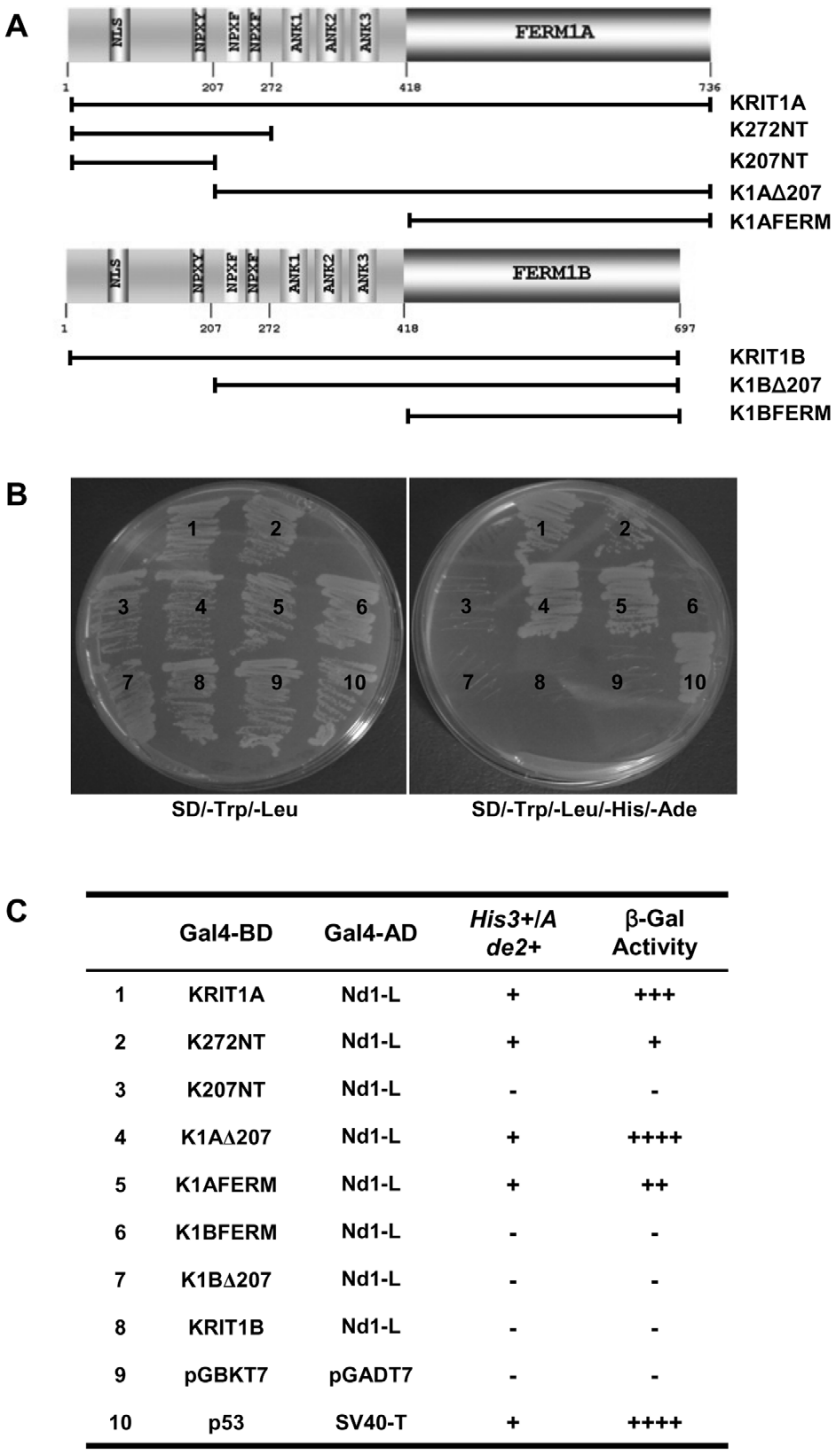
The interaction with Nd1-L involves two different regions of KRIT1. **A.** Schematic representation of the KRIT1 isoforms and deletion mutants used in the assay. **B.** Yeast two-hybrid analysis of the KRIT1-Nd1-L interaction. pGADT7-Nd1-L was used to co-transform AH109 yeast strain along with different fragments of KRIT1A and KRIT1B isoforms cloned in pGBKT7, as summarized in the associated table (**C**). Co-transformants were selected on minimal medium lacking Trp and Leu. Protein-protein interaction was assayed on the basis of the ability of Trp^+^Leu^+^ transformants to grow in minimal medium lacking His and Ade (activation of *HIS3* and *ADE2* reporter genes). Yeast cells co-transformed with the pGBKT7 and pGADT7 empty vectors were used as negative control. The interaction between SV40 large T antigen (SV40-T) and p53 was used as positive control. The results of the determination of β-galactosidase activity of Trp^+^Leu^+^His^+^ colonies by filter assay are shown in **C**. ++++ indicates blue staining of colonies detected within 3 h; +++ blue staining after 4 h; ++ blue staining after 4,5 h; + blue staining after 5,5 h; – no blue staining after 12 h. The results are representative of three independent experiments.

To better determine the contribution of the different KRIT1 domains to the interaction with Nd1-L, we assayed the ability of different regions of KRIT1 to bind full-length Nd1-L by using a small scale Y2H assay. AH109 yeast strain was co-transformed with a GAL4AD fusion construct encoding full-length Nd1-L and GAL4DNABD fusion constructs encoding different fragments of KRIT1A and KRIT1B isoforms (Fig. 5A). Yeast cells co-transformed with the two pGBKT7 and pGADT7 empty vectors were used in the Y2H experiment as negative controls to exclude any intrinsic basal transcriptional activity of the proteins used in the assay, while the interaction between SV40 large T antigen (SV40-T) and p53 was used as positive control [27]. Preliminary control co-transformations with appropriate combinations of empty vectors (pGBKT7 or pGADT7) and pGBKT7(BD)/KRIT1 or pGADT7(AD)/Nd1-L constructs, as well as Western blotting analysis of GAL4BD and GAL4AD fusion protein expression in transformed yeast extracts were also performed, and not shown for simplicity. Protein-protein interactions were assayed on the basis of the ability of transformants to grow in minimal medium lacking His and Ade (activation of *HIS3* and *ADE2* reporter genes) (Fig. 5B). Activation of the *LacZ* reporter gene was afterwards assayed by determining β-galactosidase activity of His^+^ trasformants using a filter assay.

The outcomes of these experiments are summarized in Fig. 5C. Consistently with previous results, full-length KRIT1A and the two fragments K272NT and K1AFERM, but not full-length KRIT1B and K1BFERM, were able to interact with Nd1-L in yeast cells. The KRIT1A fragment lacking the N-terminal 207 amino acids (K1AΔ207) maintained the capability to bind Nd1-L, while the corresponding K1BΔ207 fragment was devoid of this capacity. The N-terminal 207aa fragment (K207NT) did not interact with Nd1-L as well.

These data indicate that the first 207 amino acids of KRIT1 are not sufficient for the interaction of K272NT with Nd1-L, and suggest that the region 207–272 is necessary for the interaction, but not sufficient if the C-terminal portion of the protein contains an altered FERM domain.

### Nd1-L overexpression induces KRIT1 nucleus-to-cytoplasm translocation

As a first step in understanding the functional significance of the molecular interaction between Nd1-L and KRIT1, we examined the subcellular distribution of the two proteins expressed transiently in COS and A7r5 cells as fusion proteins with the C-terminus of FLAG and GFP tags, respectively. Both COS and A7r5 cells have abundant cytoplasm and hence allow easy assessment of subcellular localization.

**Figure 6:**
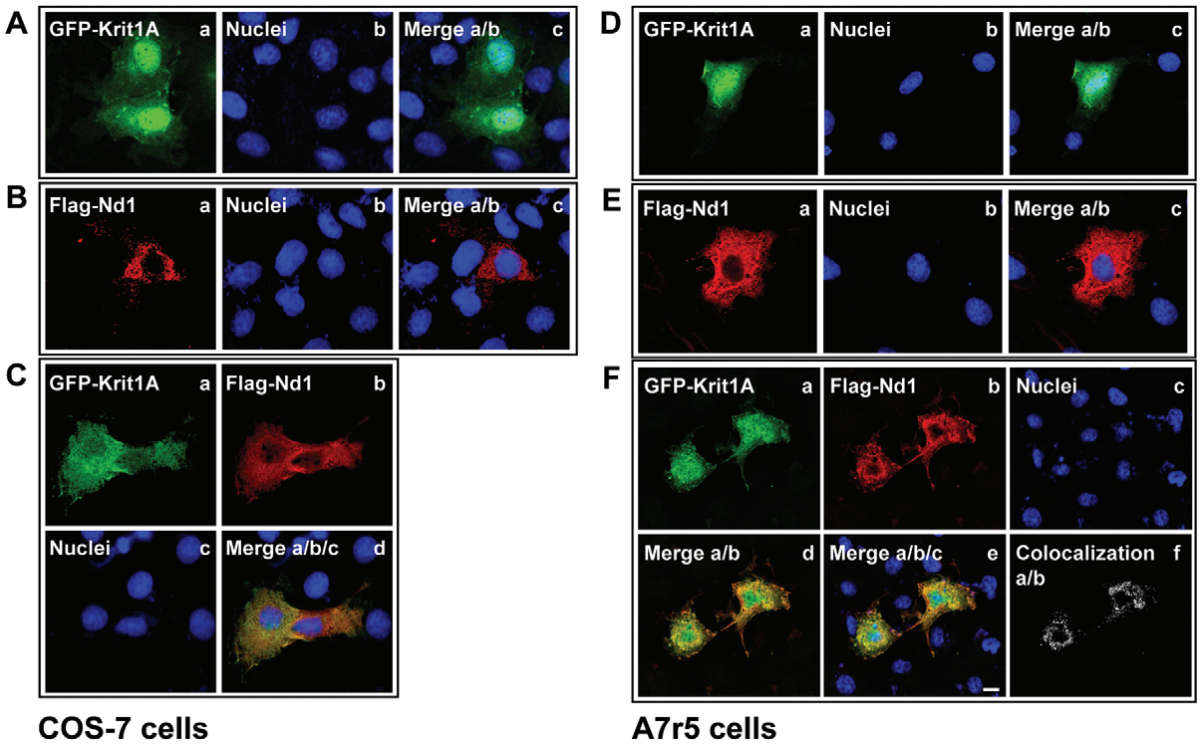
Nd1-L influences the subcellular localization of KRIT1. Confocal microscopy analysis of COS-7 (A–C) and A7r5 (D–F) cells transiently transfected with EGFP-KRIT1A (A,D) and Flag-Nd1 (B,E), or both (C,F) cDNA constructs. Nuclei and Flag-Nd1 were visualized with the Hoechst dye and anti-Flag antibody coupled to Alexa Fluor® 633 secondary antibody, respectively. Notice that GFP-KRIT1A is mainly localized into the nucleus when expressed alone (A,D), while it accumulates into the cytoplasm upon co-expression with Flag-Nd1 (C,F). Within the cytoplasm, a significant co-localization of the GFP-KRIT1A and Flag-Nd1-L proteins is also evident. Scale bar represents 15 μm.

As compared with the single-expressed GFP-KRIT1A and FLAG-Nd1-L proteins, having respectively a predominantly nuclear (Fig. 6 A,D) and a prevalently cytoplasmic (Fig. 6 B,E) subcellular distribution, the co-expression of FLAG-Nd1-L and GFP-KRIT1A resulted in a reduced nuclear accumulation of GFP-KRIT1A and a significant cytoplasmic co-localization of the two proteins (Fig. 6 C,F), indicating that Nd1-L may influence the subcellular distribution of KRIT1.

### Nd1-L may cooperate with KRIT1 in modulating the expression levels of SOD2

**Figure 7:**
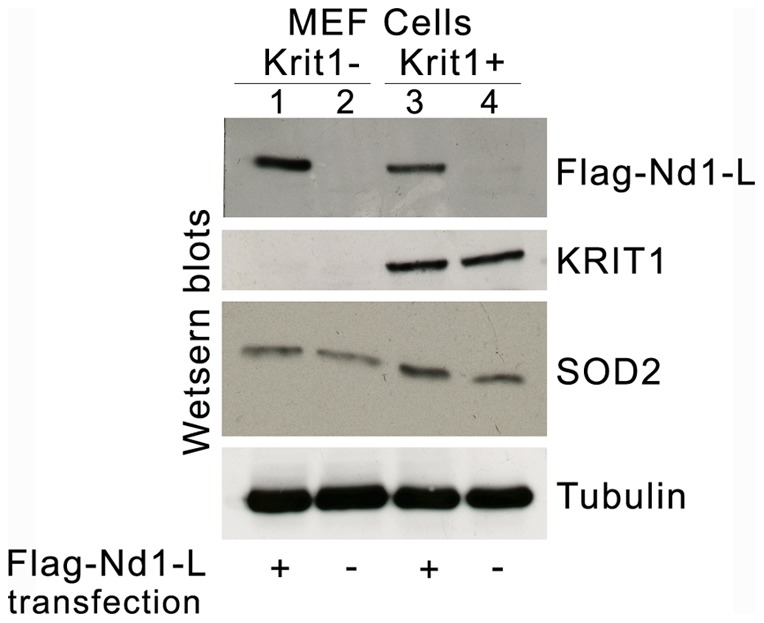
Nd1-L may cooperate with KRIT1 in modulating the expression levels of SOD2. KRIT1-null (Krit1-) and KRIT1-expressing (Krit1+) MEF cells grown under standard conditions were either mock transfected (−) or transfected with Flag-Nd1-L cDNA (+), and cell lysates were analyzed by Western blot. The expression levels of Flag-Nd1-L were assessed with an antibody against the Flag tag, whereas SOD2 and KRIT1 were detected with specific monoclonal and polyclonal antibodies, respectively. Tubulin served as loading control. Notice that the expression of Flag-Nd1-L induces an upregulation of SOD2 protein levels in KRIT1-expressing but not KRIT1-null MEF cells.

To explore further the potential functional relationship between Nd1-L and KRIT1, we took advantage of KRIT1-null and KRIT1-expressing MEF cells previously obtained and characterized in our laboratory [9]. In particular, using these cellular models we previously showed that KRIT1 plays a role in regulating the expression level of the antioxidant enzyme SOD2 [9]. To assess whether Nd1-L was able to affect this KRIT1 function, SOD2 expression levels were analyzed by western blotting in KRIT1-null (Krit1-) and KRIT1-expressing (Krit1+) MEF cells upon transient transfection with Flag-Nd1-L cDNA. Remarkably, as compared with KRIT1-null MEF cells where SOD2 protein levels were not influenced by the expression of FLAG-Nd1-L (Fig. 7, lanes 1 and 2), KRIT1-expressing MEF cells showed enhanced SOD2 levels in the presence of FLAG-Nd1-L (Fig. 7, lanes 3 and 4), suggesting a potential cooperation between Nd1-L and KRIT1 in regulating SOD2 expression, and opening a novel perspective for future mechanistic studies on the functional significance of the newly identified molecular interaction.

## Discussion

To date, the physiological function of KRIT1 is still elusive and remains a fundamental research challenge for understanding the molecular mechanisms of CCM pathogenesis. The presence in the protein of well characterized motifs and structural domains as well as its known molecular interactions [12], [13], [14], [15], [16] suggest that KRIT1 might act as a scaffold for the assembly of functional protein complexes involved in physiologically important signaling networks, including the crosstalk between cell adhesion receptors and the cytoskeleton [18].

In this study we describe the identification of the Kelch family member Nd1-L as a novel KRIT1 interacting partner.

The *Nd1* gene was originally isolated in murine cells and tissues as a housekeeping gene coding a cytosolic, actin-binding protein involved in the dynamic organization of the actin cytoskeleton [26], [28]. Further studies performed in transgenic mice indicated that the Nd1-L protein plays a protective role against cytoskeletal stress induced by doxorubicin treatment and may protect cells against oxidative stress [29], [30]. Moreover, a recent paper showed that this protein is able to negatively regulate Rho family GTPase activation, which may result in suppression of invasion and migration activity in cancer cells, thus pointing to an important functional connection between Nd1-L and Rho family proteins [31].

The human orthologue of Nd1-L, NS1-BP, has been isolated as an interactor of the nonstructural NS1 protein of the influenza A virus [32]. Although this protein has been originally shown to have a primary nuclear localization, further evidence demonstrated that also NS1-BP is able to associate with actin filaments [33].

Nd1-L contains a BTB/POZ domain in its N-terminus and six kelch repeats in the C-terminus [26]. The kelch motif appears in many different polypeptide contexts and contains multiple potential protein-protein contact sites. Members of the kelch repeat superfamily are present throughout the cell and extracellularly and have diverse activities [34]. Many kelch domain containing proteins, including Nd1-L, interact with actin and have been demonstrated to be important mediators of fundamental cellular functions, such as regulation of cellular architecture, cellular organization, and cell migration [26], [34]. In addition to the kelch motifs, Nd1-L, like the majority of the kelch proteins, contains a BTB/POZ (broad complex, tramtrack and brick a brac/Pox virus and zinc finger) domain that mediate protein-protein interactions [35], [36], and a BACK (BTB and C-terminal Kelch) domain that is thought to play a role in molecular orientation processes preliminary to protein complex formation [36].

The novel interaction between KRIT1 and Nd1-L has been identified through a Y2H screening of a mouse embryo cDNA library using the N-terminal 272 aa fragment of KRIT1 as bait. Specifically, the mouse Nd1-L cDNA isolated by the Y2H screening encoded a C-terminal 147-amino acid fragment of Nd1-L (from 496 to 642 aa residues) containing a portion of the kelch domain extending from the C-terminal portion of the third to the sixth kelch repeat motif, suggesting that this region contains the binding site for KRIT1.

The physical interaction of both the C-terminal fragment and full-length Nd1-L with KRIT1 was afterwards demonstrated using an *in vitro* GST pull-down assays, while co-IP experiments unequivocally demonstrated the association of Nd1-L and KRIT1 endogenous proteins in human endothelial cells.

Furthermore, using KRIT1A and KRIT1B isoforms and distinct KRIT1 mutated constructs we defined the contribution of the different KRIT1 domains to the KRIT1/Nd1-L interaction. We demonstrated that the KRIT1B isoform, bearing an incomplete F3/PTB FERM subdomain, is not able to bind Nd1-L, as well as that the interaction involves both N-terminal and C-terminal regions of KRIT1A.

The 272 aa N-terminal fragment of KRIT1 used as bait in the initial two-hybrid screening contains a NPXY motif required for KRIT1 interaction with ICAP1 [13], [15] and two additional NPXY/F motifs required for the interaction with CCM2 [14], [16]. In addition, the region containing the second and third NPXY/F motifs has been demonstrated to be involved in the intramolecular interaction with the PTB-like subdomain of the C-terminal FERM domain, playing a pivotal regulatory role in the control of the KRIT1A open/closed conformation switch and nucleocytoplasmic shuttling [11]. Our Y2H data indicate that the region 207–272, containing the second and third NPXY/F motifs, is necessary for the interaction with Nd1-L. However, in the full length KRIT1 protein this region is not sufficient for the interaction in the absence of an intact FERM domain, thus strengthening the active involvement of the F3/PTB FERM subdomain and consistenting with a bipartite interaction model.

Accordingly, the bipartite mode of interaction between KRIT1 and Nd1-L is reminiscent of the well characterized bipartite interaction between the FERM domain containing adaptor protein Talin and integrin β-subunit cytoplasmic tails [37], [38], which plays a major role in regulating integrin affinity for extracellular ligands [39], [40] and coupling to the actin cytoskeleton [41], [42]. Indeed, both the N-terminal FERM and the C-terminal rod domains of Talin have been found to interact with the cytoplasmic tail of integrin β-subunits. Specifically, the F3/PTB-like subdomain of the Talin FERM domain binds to a canonical NPXY motif at the membrane-proximal region of the integrin β-subunit cytoplasmic tail, whereas the integrin-binding site located in the Talin rod domain (IBS2) binds to a more distal region [37]. Interestingly, Talin may also undergo an autoinhibitory intramolecular interaction involving its N- and C-terminal portions whereby binding sites for interactors are masked. Remarkably, this molecular behavior is common to most FERM proteins, including KRIT1 [11].

Taken together with these analogies, our results point to a model where both the N- and C-terminal portions of KRIT1 are involved in the molecular interaction with Nd1-L, which in turn may contribute to maintaining the KRIT1 open conformation required for its shuttling between nucleus and cytoplasm and functioning as a scaffolding protein [11]. The bipartite mode of interaction between KRIT1 and Nd1-L might in fact compete with the KRIT1 autoinhibitory intramolecular interaction between the N- and C-terminal regions leading to KRIT1 cytoplasmic localization, which is in turn required for interaction with Rap1.

Consistently, fluorescence microscopy experiments indicated that Nd1-L contributes to the regulation of KRIT1 nucleocytoplasmic shuttling, thus providing a first evidence of the functional significance of the KRIT1A/Nd1-L interaction. In addition, molecular assays showed that Nd1-L might support KRIT1 in regulating the expression levels of the ROS-scavenging enzyme SOD2, suggesting a potential important functional cooperation in cellular protection from oxidative stress that deserves further exploration and mechanistic studies.

Thus, while showing the identification and characterization of the novel molecular interaction between KRIT1 and Nd1-L, our findings provide a promising framework for better understanding of KRIT1 physiopathological functions and addressing molecular mechanisms involved in CCM disease pathogenesis.

## Materials and Methods

### Plasmid construction

Full-length KRIT1A and KRIT1B, the K207NT (residues 1–207) and K272NT (residues 1–272) KRIT1 N-terminal fragments, the N-terminal truncated portion of KRIT1A (K1AD207; 208–736) and KRIT1B (K1BD207; 208–697) were cloned as EcoRI/SalI inserts into pGBKT7 (*TRP1, Kan^r^*) (Clontech) in frame with the GAL4DNA-binding-domain (GAL4BD).

The constructs pGBKT7-K1AFERM and pGBKT7-K1BFERM, containing the FERM domain of KRIT1A (418–736) and KRIT1B (418–697) respectively, were prepared as previously described [11].

The expression plasmid pCR-2FLAG-Nd1-L, containing murine full-length Nd1-L cDNA, was kindly provided by Dr. M. Hatano, Chiba University, Japan [26].

Full length Nd1-L was obtained from pCR-2FLAG-Nd1-L by enzymatic digestion and cloned as EcoRI/EcoRI insert into pGADT7 (*LEU2, Amp^r^*) (Clontech) vector in frame with the GAL4 Activation Domain (GAL4AD).

To generate GST-fusion proteins, full length KRIT1A, K1AFERM, K1BFERM and K272NT were cloned as EcoRI/XhoI inserts in the pGEX-4T1 vector. The expression construct encoding GST-tagged Nd1-L (496–642) was produced by EcoRI/XhoI digesting the pACT2 construct isolated by two-hybrid screening and then cloning the insert into the pGEX-4T2 vector.

Plasmid expression constructs encoding EGFP-tagged KRIT1A and KRIT1B used for cell transfection experiments were prepared as previously described [11].

The EGFP-tagged construct encoding K272NT was produced by cloning the corresponding cDNA into the EcoRI/SalI sites of pEGFP-C2 vector.

All constructed plasmids were verified by sequencing.

### Yeast two-hybrid analysis

For Y2H experiments, all vectors, yeast strains, reagents and methods were derived from the GAL4 based MATCHMAKER Two-hybrid system 3 (Clontech).

The expression of GAL4BD and GAL4AD fusion proteins used in Y2H experiments was verified by Western blotting analysis of transformed yeast protein extracts with anti-GAL4-DNA-BD or anti-HA epitope mAbs respectively.

For initial screening, the K272NT fragment fused with GAL4BD in the pGBKT7 vector was used as bait and transformed into AH109 (MATa) yeast strain, which was then mated with Y187 (MATa) yeast strain pre-transformed with a mouse embryo cDNA library fused with GAL4AD in the pACT2 vector. Diploid cells, containing the reporter genes *HIS3, ADE2, MEL1* and *LACZ*, were plated on selective medium. The clones resulting positive by the 4 reporter genes expression were candidates for harboring interacting hybrid proteins. The pACT vectors carrying potential positive interacting cDNAs were rescued from yeast cells and reintroduced into pGBKT7-K272NT- or pGBKT7-containing AH109 cells to verify the specific interaction between bait and prey proteins. Those cDNAs that exhibited a K272NT-dependent *HIS3/ADE2*-positive genotype upon retransformation were sequenced by using a GAL4AD sequencing primer and compared with known sequences in GenBank. The sequence of clone C17, studied in this work, was aligned with the nucleotide sequence NM_001039512.

For small-scale assays, pGBKT7 and pGADT7 constructs were cotransformed into AH109 cells and cotransformants were than selected on minimal medium lacking Trp and Leu. Protein-protein interactions were assayed on the basis of the ability of transformants to activate *HIS3*, *ADE2* and *LACZ* reporter genes. AH109 cells cotrasformed with pGBKT7-53, encoding a GAL4DNA-BD fusion with the murine p53 protein, and pTD1, encoding a GAL4AD fusion with the SV40 large T antigen, were used as positive controls, whereas yeast cells cotransformed with empty pGBKT7 and pGADT7 vectors were used as negative controls.

To evaluate the β-galactosidase activity of transformants a colony lift filter assay was performed by following the manufacturer instructions (Clontech). Briefly, transformants were grown on selective plates lacking Trp, Leu and His for 4–5 days; Trp+Leu+His+ colonies were transferred onto Wathman 3MM paper and immersed in liquid nitrogen for 20 sec. The filters were thawed at room temperature, overlaid with Z buffer/X-gal solution (PBS buffer, pH 7.0, 0.27% (v/v) β-mercaptoethanol, 0.03% (w/v) X-gal), incubated at room temperature and checked periodically for the appearance of blue colonies.

### Expression and purification of GST-fusion proteins

The different GST-tagged proteins were expressed and purified by using the GST Gene Fusion System (GE Healthcare). Induction was performed by incubating transformed *E.coli* BL21 cells at 18–30°C with IPTG (0.1–0.2 mM). GST fusion proteins were finally analyzed by western blotting with anti-GST antibody (GE Healthcare). The amount of expressed proteins was determined by using the Bradford method [43].

### Cell culture and transient transfections

The following cell lines were used: HEK293 human embryonic kidney cells and COS-7 monkey kidney fibroblast cells, purchased from LG Standards-ATCC, UK (CRL-1573 and CRL-1651 respectively); A7r5 rat vascular smooth muscle cells, purchased from Sigma-Aldrich, Italy (8605803); KRIT1^−/−^ MEFs (K^−/−^) and KRIT1^−/−^ MEFs re-expressing KRIT1 (K9/6), described previously [9].

All these cell lines were cultured at 37°C and 5% CO_2_ in DMEM supplemented with 10% FBS, 1% penicillin/streptomycin, 2 mM glutamine (only COS-7 and A7r5 cells). All media and supplements for cell culture were from Gibco Life Technologies.

Human Umbilical Vein Endothelial Cells (HUVECs), purchased from Lonza (CC-2519, Lonza Group Ltd, Switzerland), were cultured on gelatine-coated dishes in M199 medium (Sigma) supplemented with 10% FCS, 10 μg/ml heparin, endothelial cell growth supplement (ECGS, Sigma), glutamine and antibiotics. Each culture was used only up to eight population doublings.

For transient transfection of HEK293 cells to be used in GST-pull down and co-IP experiments, cells were plated one day before transfection and grown to semiconfluence. Transfections were carried out using the calcium-phosphate method and assays were performed 36 h after transfection.

COS-7 and A7r5 cells to be used for fluorescence microscopy analysis were transiently transfected with EGFP-KRIT1A and Flag-Nd1-L cDNAs using FuGENE 6 (Roche) and TransIT®-2020 (Mirus) transfection reagents, respectively, according to the manufacturers’ instructions. The same method was used to transiently transfect K^−/−^ and K9/6 MEF cells [9] with Flag-Nd1-L cDNA for Western blotting analyses.

### Fluorescence microscopy

48 h after transfection, COS-7 or A7r5 cells were fixed in 3,7% paraformaldehyde for 15 min. Cells expressing Flag-Nd1-L were than permeabilized with 0.5% Triton X-100 in TBS for 1 min, incubated with 1% BSA in TBS for 1 h, and stained with anti-Flag epitope primary antibody (F3165, Sigma) and Alexa Fluor® 633 (Invitrogen) secondary antibodies. All transfected cells were then counterstained with a blue fluorescent nuclear dye (Hoechst 34580) and mounted on microscope slides with ProLongH Gold antifade reagent (Molecular Probes, Invitrogen) before imaging. Confocal microscopy imaging was performed on a Leica TCS_SP5 confocal microscope (Leica Microsystems) using a PlanApo 63x/1.40 oil immersion objective. Instrument parameters for sequential image acquisition, including pinhole diameter, laser intensity, exposure time, PMT gain and offset, were set and held constant to minimize autofluorescence and for comparison between samples. Colocalization images were obtained using the ImageJ software.

### Cell lysates

To prepare cell lysates, transfected or mock-transfected HEK293, HUVEC or MEF cells in 90 mm plates were washed with ice-cold PBS, scraped in the presence of NP-40 lysis buffer containing protease inhibitors (KIT Complete, Roche), collected in a clean tube and finally incubated on ice for 20 min. The cell lysates were centrifuged for 10 min at 15000×g and the supernatant was collected in a clean tube for further analysis.

### GST-Pull-down experiments

GST fusion proteins (40 µg) immobilized on 40 µl of glutathione-sepharose 4B beads (50% in PBS) were incubated for 1 h at room temperature with of HEK293 cells lysates overexpressing the GFP- or 2FLAG-tagged target proteins. The beads were subsequently washed three times with PBS buffer, added with SDS gel-loading buffer and finally analyzed by SDS-PAGE followed by anti-GFP (Sigma) or anti-FLAG (Sigma) western blotting.

### Co-immunoprecipitation (co-IP) experiments

For co-IP of endogenous proteins (KRIT1 and Nd1-L) HUVEC cell lysates were prepared as described and then pre-cleared by adding 20 µl of Protein A/agarose Fast-flow (Sigma) (50%) per 500 µg of cell lysate and incubated at 4°C for 1 h in an orbital shaker. The protein A was removed by centrifugation and the supernatant was incubated overnight at 4°C with a polyclonal anti-KRIT1 antibody produced in our laboratory. In order to capture the immunocomplex, 30 µl of ProteinA/agarose (50%) were added to the incubation mix and incubated at 4°C for 4 h. The agarose slurry was collected by centrifugation and the supernatant was discarded; the pellet was washed three times with NP-40 lysis buffer, then resuspended in SDS gel-loading buffer and analyzed by SDS-PAGE followed by anti-Nd1-L (Sigma) western blotting.

For co-IP of overexpressed proteins, lysates from HEK293 cells co-transfected with the expression vectors encoding EGFP-tagged KRIT1A or KRIT1B and 2FLAG-Nd1-L were used. The co-immunoprecipation was performed as above by using anti-KRIT1 to precipitate the immunocomplex and anti-FLAG to perform western blotting analysis.

### Western blotting

Western blotting analyses were performed by using the Snap i.d. Protein Detection System (Millipore, WBAV DBA SE). Briefly, the different samples were separated by SDS-PAGE and electrotransferred onto nitrocellulose membranes. The blots were then assembled in the blot holder and treated according to the manufacturer’s instructions using distinct primary antibodies, including mouse mAbs against SOD2 (16956, Abcam) and Tubulin (T5168, Sigma). Primary antibodies were detected using affinity purified HRP-conjugated secondary antibodies (Sigma). Immunoreactive proteins were finally visualized by an enhanced chemioluminescence (ECL) detection system (GE Healthcare).

The results showed in the Figures are representative of at least three independent experiments.
